# Potential Challenges for Coronavirus (SARS-CoV-2) Vaccines Under Trial

**DOI:** 10.3389/fimmu.2020.561851

**Published:** 2020-09-29

**Authors:** Salman Sadullah Usmani, Gajendra P. S. Raghava

**Affiliations:** Department of Computational Biology, Indraprastha Institute of Information Technology, New Delhi, India

**Keywords:** coronavirus, COVID-19, SARS-CoV-2, vaccine candidates, immunoinformatic

## Introduction

Many vaccines are under clinical trials to fight against pandemic COVID-19. Here, we briefly reviewed vaccines under trial and their limitations, with a possible alternate solution to overcome these limitations. In most cases, vaccine targets are all viral proteins or a specific protein, mainly spike protein. As shown in previous studies on coronavirus strains, these vaccine targets may cause a wide range of side effects. These include the induction of cytokine storms (i.e., IL6), lung immunopathology, hepatitis, hemotoxicity, cytotoxicity, cross-reactive antibodies, allergenicity. Minimizing the size of vaccine candidates from protein to epitope/peptide will overcome the side effects. In our opinion, there is a need to utilize computer-aided techniques for the identification of potential vaccine candidates to fight against COVID-19.

The pandemic COVID-19, a severe acute respiratory syndrome coronavirus 2 (SARS-CoV-2) disease, emerged as the most recent significant challenge to global health and prosperity since World War II. WHO has reported more than 26 million cases, with 0.87 million deaths up to September 6, 2020 (1). Lack of proper medication forced many countries to opt for complete lockdown, which caused chaos in the economy (2). COVID-19 posed a threat not only to lives but also to freedom, it threatened to tear the world into boundaries and lock people into confined regions. An exponential increase in the infected cases forced the researcher to look up for the complete cure as early as possible. Thus, the COVID-19 vaccine has become a holy grail. At the start of May 2020, more than 120 vaccines were in the pipeline throughout the world, and at least six groups had started injecting the formulation into volunteers (3). According to WHO, 34 vaccine candidates were in clinical evaluation, among which eight candidates entered into phase 3 of the clinical trial as of September 3, 2020. Besides this, about 142 vaccines are in preclinical evaluation (4).

## An Overview of Current Vaccine Trials

**Figure 1:**
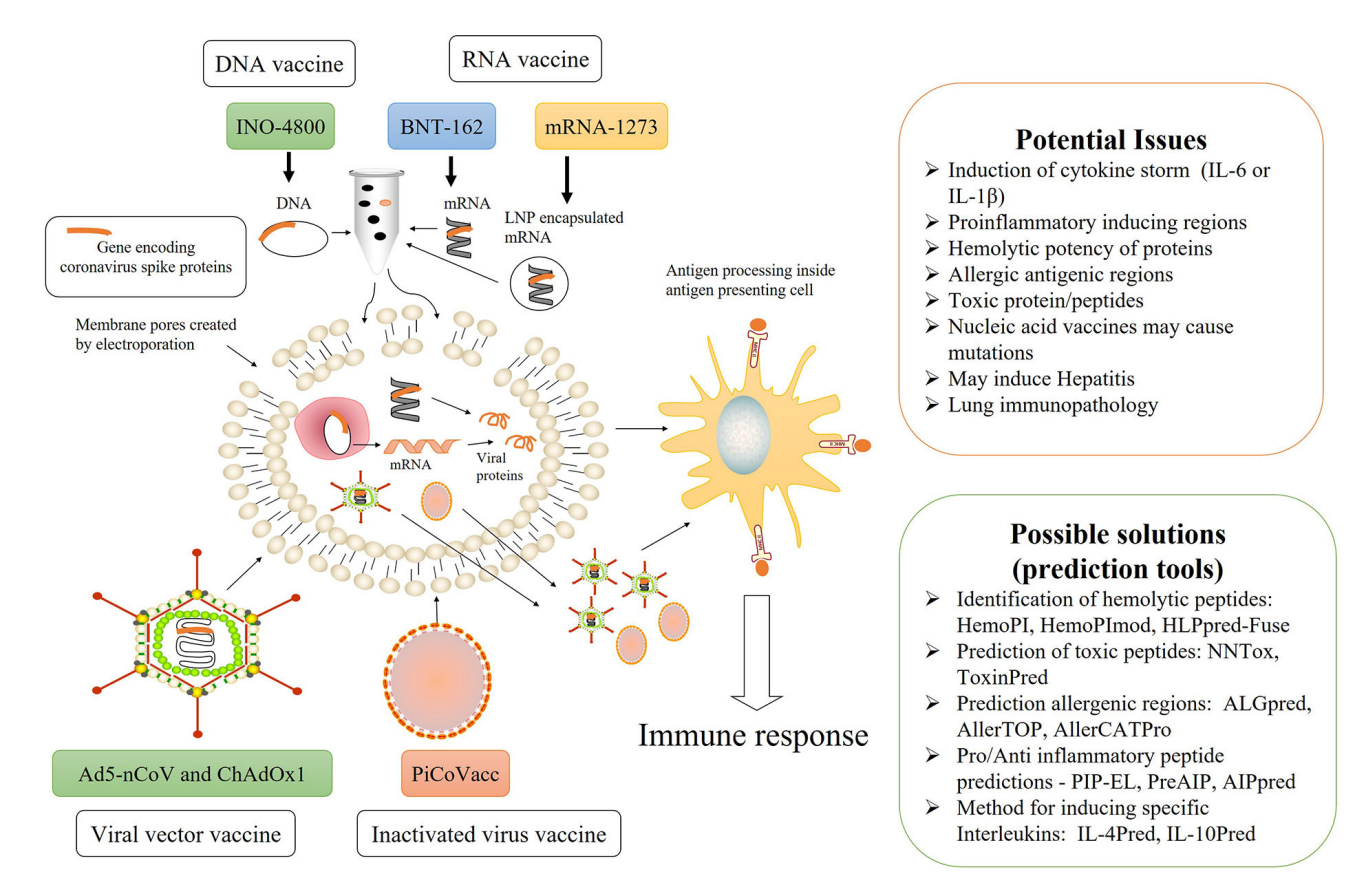
Overview of current SARS-CoV-2 vaccines under trial, their possible limitations, and computer-aided solutions.

Ad5-nCoV, developed by CanSino Biologics and an arm of the People’s Liberation Army, was the first candidate to enter human trials and is currently in clinical phase 2. It is a viral vector vaccine and uses non-replicating adenovirus type 5 vector, to transport the DNA of spike proteins of SARS-CoV-2 (3, 5). The concept is to transform the spike proteins within the body, ultimately leading to the activation of the immune system. Using the same technology, researchers from Oxford University developed ChAdOx1, which will eventually produce spike proteins within the human body, leading to immune system activation. ChAdOx1 has already started phase 3 clinical trials, after showing an acceptable safety profile in single blind, randomized phase 1/2 clinical trials (6). Inovio Pharmaceuticals, a US-based biotechnology firm, has developed INO-4800, a DNA-based vaccine, utilizing a relatively newer vaccine technique. Here, DNA containing the genetic code of SARS-CoV-2 spike proteins will be injected into human cells by electroporation devices, and the transformed spike proteins will activate the immune system. PiCoVacc, currently in phase 3 trial, is an inactivated vaccine developed by Sinovac, a Chinese private biopharma company, based on the traditional fact that exposure to an inactivated virus will eventually lead up to the immune response (7). The Beijing Institute of Biological Products/Wuhan Institute of Biological Products is working on few inactivated vaccines, which are in the clinical phases 2/3. However, they are relatively unadvertised outside Chinese media (8). Besides this, two RNA vaccines are also in the pipeline, named as mRNA-1273 by US biotech firm Moderna/NIAID and BNT-162 jointly by German company BioNTech and US pharma giant Pfizer. Both these vaccines follow the concept of delivering information molecules to instruct human body cells to produce the spike proteins of SARS-CoV-2. This information molecule is mRNA in the case of BNT-162, whereas LNP-encapsulated mRNA in mRNA-1273 (9, 10) (**Figure 1**).

## Revaluating Vaccines for Previous Coronavirus Strains

The current distressing worldwide scenario demands a vaccine at the earliest, causing a race among pharmaceutical industries. This accelerated version of vaccine development implements parallel steps like animal testing, clinical phase 1, or simultaneous phase 1 and 2 clinical trials. By involving several manufacturers, the developers of ChAdOx1 have already planned to produce a million-dose by September 2020 (11). However, the question is whether speeding the trail is safe. What are the possibilities of getting a vaccine with the highest efficacy concerning diverse ages and ethnicity? What is the financial risk in scaling up production capacity, even before sufficient efficacy data, like in the case of ChAdOx1? These questions make sense if we re-evaluate recent virus outbreaks, including Ebola, Zika, or earlier coronaviruses, i.e., SARS and MERS. The Ebola and Zika epidemics ended before vaccine development, leaving the manufacturer with financial loss due to the reallocation of funds by federal agencies (12). The SARS (severe acute respiratory syndrome) coronavirus emerged in 2002-03 and affected 26 countries, whereas the MERS (Middle East respiratory syndrome) coronavirus emerged in 2012 and affected 27 countries (13). However, the astonishing fact is that no vaccine has been commercially available for these coronaviruses until now (14). Inactivated SARS virus (15), live attenuated/host-adapted SARS virus (16), replicating and non-replicating viral vector (17, 18), DNA based vaccine (19), soluble proteins/adjuvants (20), virus-like peptide/adjuvants (21), and a combination of vaccines approach (22, 23) have been used to develop a vaccine against SARS-CoV, but to the best of our knowledge only inactivated SARS virus, DNA and soluble SARS S-glycoprotein reached the clinical phase 1 trials (14). Similarly, for MERS, several vaccines have been developed, but only a DNA based vaccine, targeting S-glycoproteins and subunits, is in a clinical phase 1 trial (24). The vaccine development might have been slowed by several reasons such as a lack of suitable animal models, as they exhibited limited viral replication and clinical manifestations (25) or geographically centralized cases might have declined the interest of pharmaceutical companies. Whatever the reason for the lack of vaccine or financial losses, there is a need to revisit these cases while investing in the development of the SARS-CoV-2 vaccine.

## Challenge: Balancing Immune Response

The nature of COVID-19 transmission makes it more devastating, as the median incubation time from infection to symptoms ranges from 4-7 days. Besides this, many infected patients remain asymptomatic but prone to transmit the virus (26). It is quite evident that both innate and adaptive immunity plays a role during the COVID-19 infection, but how their interaction mediates viral control as well as host toxicity, is not very clear yet. Based on pieces of evidence, it has been suggested that an innate immune-mediated ‘cytokine storm’ is responsible for toxicity and organ damage in a subgroup of patients with a severe COVID-19 infection (27). Extended populations of IL-6 and IL-1β secreting monocytes have been found in COVID-19 infected patients, resulting in elevated serum IL-6 and lactate dehydrogenase, a marker of pyroptosis, a highly inflammatory form of programmed cell death (28, 29). Similarly, an elevated level of ferritin and IL-6 in 150 multicenter COVID-19 confirmed cases suggested that virally driven hyper-inflammation might be the reason for mortality (30). It is a known fact that virally derived pathogen and damage-associated molecular patterns (PAMP and DAMP) activate macrophages, resulting in the downstream production of IL-6 and IL-1β, which further recruits neutrophils and CD8^+^ T cells, thus controlling viral growth. However, within the lung parenchyma, neutrophils also induce tissue damage by releasing leukotrienes and reactive oxygen species, giving passage to alveolar flooding and fibrosis. In severe infection, persistent IL-6 elevation leads to constant neutrophil-mediated alveolar damage, resulting in the need for mechanical ventilation and, ultimately, mortality (31). We all know that an early innate and adaptive immune response leads to the suppression of acute viral infection. However, chronic viral infection causes T cell depletion and exhaustion, leaving persistent innate activation, triggering inflammation, and cytokine toxicity. An intact T cell-mediated immune response is the key contributor in clearing and maintaining long term suppression (32). It is a growing possibility that immunosuppression due to the depletion and exhaustion of T cells contribute to COVID-19 persistence and mortality (27, 33).

There are several studies showing a paradoxical phenomenon, in which vaccinated animals or people exhibit a more severe disease while exposed to the virus than non-activated fellows (34–36). This immune backfiring is known as antibody dependent enhancement (ADE), in which the virus influences antibodies for its own benefit and enhances the infection or immune enhancement, consisting of allergic inflammation due to Th2 immunopathology. ADE has been observed with dengue, HIV, flavivirus, as well as Zika virus (37–40). The hunt for a SARS and MERS vaccine has been stymied by ADE. It has been shown that, antibody-dependent SARS coronavirus infection is mediated by antibodies against spike proteins (41, 42). ADE or eosinophil-mediated immunopathology has been observed while immunizing mice with inactivated whole SARS-CoV (43) or DNA vaccine encoding full length S-protein (44) as well as immunizing macaque models with MVA encoded S protein (45). There have been various recently published articles concerning the potential danger of suboptimal antibody responses or ADE of SARS-CoV-2, providing an insightful discussion (46–49).

## Conclusion: An Integrated Approach Is Needed

It is obvious to consider the fact that both an innate and adaptive immunity participates in COVID-19 mediated toxicity while designing vaccines. There is always a possibility of mutation within a human genome by nucleic acid vaccines (50). What if the spike proteins contain the region which could elicit the IL-6 or IL-1β level? What if the nucleic acid fragment delivered within the body transforms into a region of spike proteins that are proinflammatory or have any other interleukin-inducing peptides? There are significant possibilities of having toxic peptide fragments in COVID-19 spike proteins. The peptide region may have hemolytic potential, resulting in hemolysis as a severe side effect. Besides this, the coronavirus spike protein may contain the regions prone to allergy, and their processing inside the cell may lead to allergic antigens. Have they been ruled out, while delivering spike protein regions into the body? Bioinformatics might provide an answer to these queries to some extent. There are several *in silico* tools to predict interleukin-inducing properties, pro/anti- inflammatory as well as the toxicity of peptides (51–60). Besides this, many *in silico* tools are available for designing subunit vaccines and immunotherapeutics (61, 62). There are 244,682 proteins and 22,892 nucleotide sequence information about SARS-CoV-2 available in the NCBI virus datahub, as of September 8, 2020 (63). It is obvious that all 244,682 proteins cannot be a vaccine candidate, so we can use various *in silico* tools to identify potential vaccine candidates. These candidates must be further scrutinized for potential epitopes, which can activate the desired arm of the immune system. The half-life, toxicity, and unwanted properties like in SARS-CoV-2 cases such as IL-6 inducing potential, immunosuppressive property, hemolysis, and allergy must also be checked. We have enlisted some useful *in silico* tools in **Figure 1**. Various researchers have already identified potential B and T cell epitopes, HLA susceptibility mapping as well as candidate targets by applying a bioinformatic approach (64–69). In the cat and mouse game of a viral pandemic and vaccine development, bioinformatic resources are beneficial and will cut down the chances of failure, a lesson by previous virus endemics. There are significant chances of severe side effects, as the frenetic race against time forced the vaccine trials on a fast track. Due to emergency and the worldwide crisis, there is a possibility of accelerating the trial mechanism by reducing numbers and waving off some of the population, as reported in several news agencies (70, 71). We hope that the devastating worldwide scenario will end up with a successful vaccine candidate soon.

## Author Contributions

SU wrote the manuscript while GR conceived the idea as well as edited the manuscript.

## Conflict of Interest

The authors declare that the research was conducted in the absence of any commercial or financial relationships that could be construed as a potential conflict of interest.
